# Estimation of acute oral toxicity in rat using local lazy learning

**DOI:** 10.1186/1758-2946-6-26

**Published:** 2014-05-16

**Authors:** Jing Lu, Jianlong Peng, Jinan Wang, Qiancheng Shen, Yi Bi, Likun Gong, Mingyue Zheng, Xiaomin Luo, Weiliang Zhu, Hualiang Jiang, Kaixian Chen

**Affiliations:** 1 Department of Medicinal Chemistry, School of Pharmacy, Yantai University, Yantai, Shandong 264005, China; 2 Drug Discovery and Design Center, State Key Laboratory of Drug Research, Shanghai Institute of Materia Medica, Chinese Academy of Sciences, 555 Zuchongzhi Road, Shanghai 201203, China; 3 School of Life Science and Technology, ShanghaiTech University, Shanghai 200031, China; 4 School of Pharmacy, East China University of Science and Technology, Shanghai 200237, China

**Keywords:** Acute toxicity, Local lazy learning, Applicability domain, Consensus model

## Abstract

**Background:**

Acute toxicity means the ability of a substance to cause adverse effects within a short period following dosing or exposure, which is usually the first step in the toxicological investigations of unknown substances. The median lethal dose, LD_50_, is frequently used as a general indicator of a substance’s acute toxicity, and there is a high demand on developing non-animal-based prediction of LD_50_. Unfortunately, it is difficult to accurately predict compound LD_50_ using a single QSAR model, because the acute toxicity may involve complex mechanisms and multiple biochemical processes.

**Results:**

In this study, we reported the use of local lazy learning (LLL) methods, which could capture subtle local structure-toxicity relationships around each query compound, to develop LD_50_ prediction models: (a) local lazy regression (LLR): a linear regression model built using *k* neighbors; (b) SA: the arithmetical mean of the activities of *k* nearest neighbors; (c) SR: the weighted mean of the activities of *k* nearest neighbors; (d) GP: the projection point of the compound on the line defined by its two nearest neighbors. We defined the applicability domain (AD) to decide to what an extent and under what circumstances the prediction is reliable. In the end, we developed a consensus model based on the predicted values of individual LLL models, yielding correlation coefficients R^2^ of 0.712 on a test set containing 2,896 compounds.

**Conclusion:**

Encouraged by the promising results, we expect that our consensus LLL model of LD_50_ would become a useful tool for predicting acute toxicity. All models developed in this study are available via http://www.dddc.ac.cn/admetus.

## Background

Estimation of rodent acute toxicity is an important task in the safety assessment of drug candidates. Median lethal dose (LD_50_), a dose causing 50% death of the treated animals in a given period when administered in an acute toxicity test [1], is a common criterion that measures acute toxicity of compound. However, due to ethical reasons, the animal experiments on rodent acute toxicity are highly controversial. European Union Registration, Evaluation, Authorization and Restriction of Chemicals (REACH) has recommended the use of *in vitro* or *in silico* methods instead of animal testing of LD_50_[2]. This proposal drives the development of quick, reliable, and non-animal predicting methods such as quantitative structure-toxicity relationships (QSTRs).

Acute toxicity involves multiple biochemical mechanisms, and a large number of compounds have been reported for their LD_50_ information, which covers a significant portion of chemical diversity space. These complexities pose a big challenge to the building of a single QSAR model with high prediction accuracy. Taking the acute rodent toxicity as an example, Enslein *et al.*[3,4] developed multiple linear regression (MLR) models based on noncongeneric datasets, and found that the models had poor prediction power. To increase the prediction accuracy, Eldred *et al.*[5] and Guo *et al.*[6] built a few local models based on congeneric datasets. This type of models has improved accuracy, but their application ranges are limited. Zhu *et al.*[7] introduced the applicability domain (AD) in their study, and constructed consensus model from multiple individual models using *k* nearest neighbors (KNN), random forest, hierarchical clustering, and so on. The consensus model showed improved results as compared to the individual constituent models, while the prediction accuracy is still limited when the model coverage increases.

Due to the complex mechanisms of acute toxicity, we explored the similarity-based local models to study the rat LD_50_ data by oral exposure. The basic idea of such models follow that “structurally similar molecules are likely to have similar properties”, which is suitable for modeling very complex boundaries between two classes [8]. In light of the idea, Yuan *et al.*[9] proposed a method “Clustering first, and then modeling”. It means the training set members are firstly grouped together based on their structural similarity. Then, the test set member is assigned to a specific group according to its structural resemblance to the group members, and its toxicity value is next predicted using an on-the-fly constructed model from the group. This method shows good performance for the datasets with distinct clusters, but it has the disadvantage of requiring *a priori* knowledge of the number of clusters. In this study, we try to use local lazy learning (LLL) to solve this problem. Given a test compound, LLL method firstly find its *k* nearest neighbors in the training set by using a predefined property set (molecular fingerprints or descriptors), and then build local models using these compounds to predict the value of the test compound. This method can fully consider the structural information of every test compound, while doesn’t rely on *a priori* knowledge of clusters. Moreover, to further improve the prediction accuracy, we try to enrich the reference data set and construct consensus models, which are critical for reducing the high variance of individual models. In the end, we analyze the application domain of the resulted models.

## Results and discussion

### Performance evaluation of LLL models

The use of LLL models makes it possible to explore many local structure-toxicity trends rather than global trends, which is expected to achieve an improvement in the prediction accuracy. Among the four types of LLL models, LLR prediction is based on a linear regression model with a single explanatory variable. In contrast, SA, SR, and GP predictions are directly based on the LD_50_ values of the query’s neighbors. In assessing molecular similarity, we used three structural (ECFP4, FCFP4, and MACCS) and descriptor-based (DES) metrics to determine which compounds would be selected as neighbors of a query from different aspects. Since each LLL model can be combined with each type of the metrics, there are 16 individual models in all. During constructing *k*NN-like models, the choice of *k* is very critical. A small value of *k* can make noises have a higher influence on the result, while a large one makes it computationally expensive and does not follow the underlying assumption that similar compounds share similar toxicity. Here the LLR and GP models automatically learn a specific *k* for each query compound. In contrast, SA and SR models use a fixed number of neighbors, which is optimized using cross-validation on the whole reference set. Table 1 summarized the statistics of the models on the test set using reference Set I, together with the best results of Zhu *et al.*[7] for comparison. Among the four LLL models, LLR has the lowest R^2^ and the largest MAE (mean absolute error), and the results of GP and SR are slightly better than SA. It is not surprising to notice that LLR yielded inferior prediction accuracy. Compared with other models, LLR has the ability to make prediction outside the range of our reference data. However, it is also subject to greater uncertainties and a higher risk of producing meaningless results, as it relies on not only the existence of similar reference compounds, but also the choice of relevant variables to establish the local regression model. For the other three methods, the SA model assumed equal contribution of all neighbors. However, when constructing similarity-based models, it cannot be guaranteed that all neighbors are similar enough to the test compound. Therefore, the performance of SR and GP is expected to be improved by applying weighting strategy such that more similar neighbors contribute more to the prediction. In this respect, we may find that the prediction accuracies of different models agree with our expectation. Moreover, for the four similarity metrics, most results obtained from fingerprints (ECFP4, FCFP4, and MACCS) were found superior to the results from DES. This observation suggests that the structural similarity is more efficient than the descriptor-based similarity in selecting neighbors with equivalent toxicity level. Among the 16 individual LLL models, the best combination is GP + ECFP4. It yielded R^2^ of 0.491 for “Set_2896”, 0.514 for “Set_2583”, and 0.719 for “Set_743”. Apart from being used to build predictive models, the fingerprints can also be analyzed to find out the fragments that may cause acute toxicity. The corresponding analysis with ECFP4 is provided in Additional file 1.

**Table 1:** Performance of four LLL models using different similarity metrics on the test set using reference set I (Group I) versus the best model of the reference

**Models**		**“Set_3874”**		**“Set_2896”**		**“Set_2583”**		**“Set_743”**	
		**R**^**2**^	**MAE**	**R**^**2**^	**MAE**	**R**^**2**^	**MAE**	**R**^**2**^	**MAE**
LLR	ECFP4	0.347	0.622	0.415	0.589	0.437	0.580	0.647	0.474
	FCFP4	0.333	0.629	0.433	0.589	0.460	0.575	0.649	0.477
	MACCS	0.317	0.640	0.386	0.608	0.407	0.599	0.644	0.506
	DES	0.255	0.667	0.348	0.608	0.381	0.588	0.578	0.444
	LLR_consensus	0.434	0.535	0.511	0.508	0.534	0.499	0.738	0.401
SA	ECFP4	0.390	0.563	0.464	0.535	0.486	0.526	0.688	0.427
	FCFP4	0.363	0.581	0.469	0.543	0.494	0.527	0.674	0.441
	MACCS	0.379	0.573	0.452	0.546	0.467	0.542	0.679	0.452
	DES	0.329	0.591	0.419	0.539	0.448	0.523	0.627	0.407
	SA_consensus	0.450	0.528	0.527	0.493	0.544	0.490	0.746	0.394
SR	ECFP4	0.406	0.555	0.483	0.524	0.508	0.514	0.719	0.408
	FCFP4	0.376	0.575	0.485	0.536	0.513	0.519	0.701	0.426
	MACCS	0.383	0.570	0.457	0.543	0.473	0.539	0.690	0.445
	DES	0.329	0.590	0.419	0.539	0.448	0.523	0.627	0.407
	SR_consensus	0.457	0.515	0.536	0.489	0.555	0.485	0.761	0.384
GP	ECFP4	0.413	0.550	0.491	0.519	0.514	0.510	0.719	0.406
	FCFP4	0.367	0.583	0.477	0.543	0.504	0.527	0.689	0.437
	MACCS	0.360	0.586	0.443	0.556	0.461	0.551	0.692	0.451
	DES	0.329	0.586	0.421	0.534	0.450	0.518	0.627	0.412
	GP_consensus	0.460	0.512	0.545	0.483	0.565	0.477	0.771	0.371
Final_consensus		0.466	0.510	0.545	0.483	0.565	0.478	0.769	0.374
Reference	Individual^a^	n/a^b^	n/a^b^	0.41	0.55	0.41	0.56	0.70	0.41
	Consensus	n/a	n/a	0.42	0.52	0.48	0.51	0.71	0.39

^a^For each coverage set, the best result among all individual models reported by Zhu et al. [7] was shown.

^b^The reference did not report the prediction results of all compounds in the test set.

### Performance improvement by constructing consensus model

**Figure 1:**
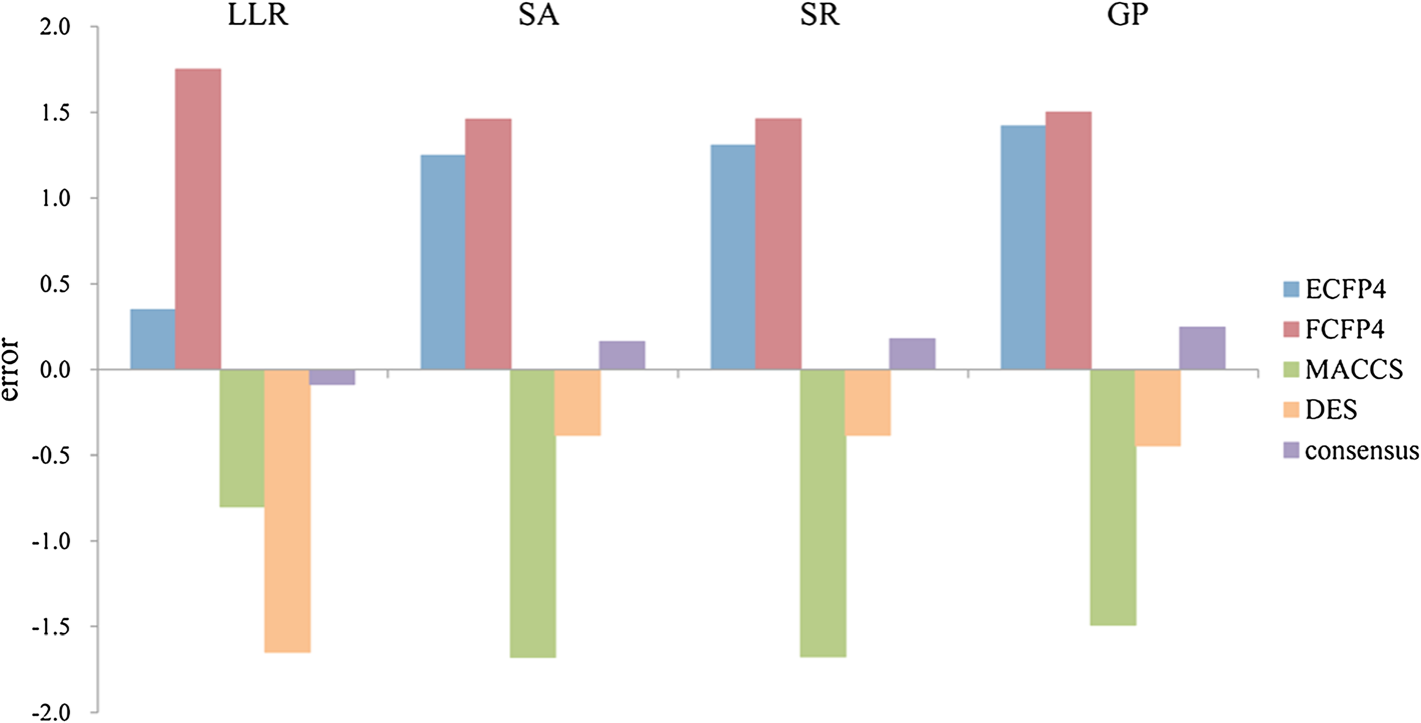
**Example to show how consensus model improve the prediction results.** Comparison of estimates for an herbicide (CAS: 89427-44-1) using individual and consensus models from Group I. The y-axis is prediction error.

LLL models using different learning algorithm or similarity metrics can possess the high variance. For example, the descriptors used in this study characterize whether two compounds share similar physic-chemical properties, while fingerprints more focus on the 2D structure similarity, leading to that an individual model could only capture part of the relationship. Depending on the analogues retrieved by different similarity metrics, the learned decision boundaries and values can vary significantly. As a result, each compound has a chance of being successfully predicted for some individual models. This can be illustrated by the prediction of 5,6,7-Trichloro-2-(trifluoromethyl)-4- benzimidazolesulfonamide [10]. As shown in Figure 1, we may notice that all the individual model predictions show great variability, in which the toxicity is overestimated by using ECFP4 and FCFP4, but is underestimated by using MACCSS and DES. Accordingly, the average prediction from individual models is closer to the actual LD_50_ value. Therefore, to explore the complementary features of different modeling techniques for predicting the acute oral toxicity, we also constructed consensus models based on the above described individual LLL models. Given a query compound, its LD_50_ value in consensus model is predicted as the arithmetic average of all LD_50_ values from individual models. The statistical results of different consensus models on the test set were listed in Table 1. Clearly, all the consensus models showed improved performance as compared to their constitutional ones. Most of the prediction accuracies of the LLR, SA, SR, and GP consensus models were higher than those of the reference (e.g., R^2^ of “Set_2896” is 0.545 for GP versus 0.42 for the reference consensus model). Of note is the final consensus model. It obtained further improved R^2^ values ranging from 0.545 to 0.769 for “Set_2896”, “Set_2583” and “Set_743”, significantly higher than those of the reference consensus model. These results demonstrated that the LLL model based averaging scheme is an efficient way for enhancing prediction accuracy of acute toxicity prediction.

### Performance improvement by enriching the reference set

**Figure 2:**
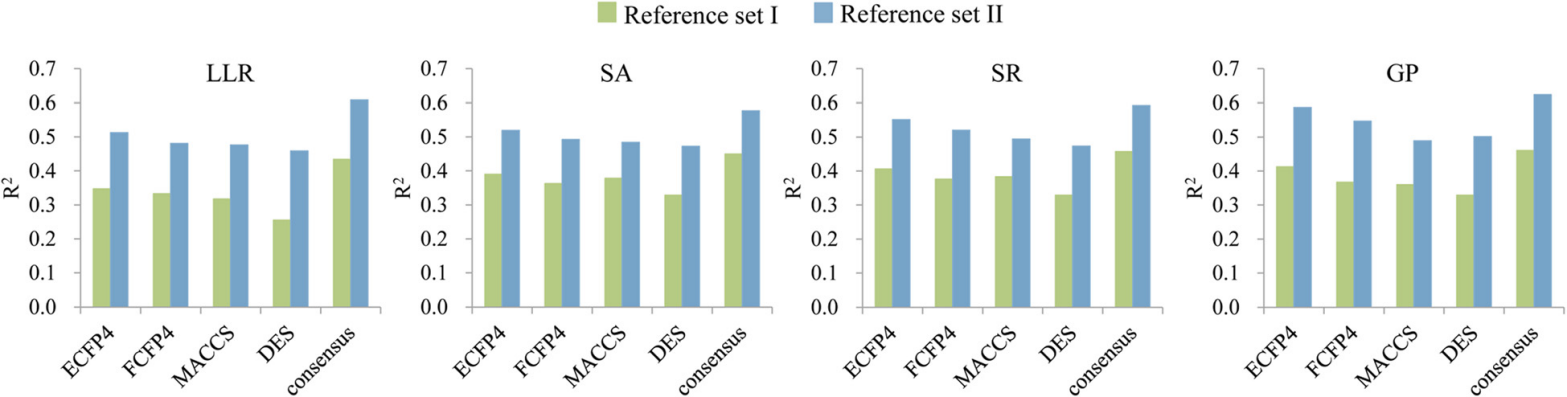
**Distribution of R**^**2 **^**values for the prediction of whole test set (“Set_3874”) using different reference set.**

The above results suggest that the use of LLL approaches can generally lead to an improvement in LD_50_ prediction accuracy. Then, we further inspected the performance of LLL models on the four different test sets. One can easily see that a significantly better prediction can be achieved for compounds which have more similar neighbors in the reference set. Clearly, a large and structurally diverse reference set is essential to the similarity based approaches. To enrich the chemical diversity space covered by the reference library, 2,271 compounds with LD_50_ values from EPA [11] and Accelrys Toxicity database [12] were combined with the reference set I to compose the reference set II. Table 2 summarized the statistics of all individual and consensus models on the test set by applying the expanded reference set. To make the comparison more clearly, we also plotted the distribution of performance on the whole test set (“Set_3874”) using the two reference set. As shown in Figure 2, we may find that the increasing of the size and diversity of reference set evidently contributed to the improvement of results. For example, the model GP + ECFP4 using reference set II obtained a R^2^ of 0.587 on the whole test set, while the corresponding model using reference set I only gave a R^2^ of 0.413. In contrast to the subtle differences among the results of various LLL schemes, the enrichment of the reference set substantially improved the prediction performance of all models.

**Table 2:** Performance of our models on the test set using the reference set II (Group II)

**Models**		**“Set_3874”**		**“Set_2896”**		**“Set_2583”**		**“Set_743”**	
		**R**^**2**^	**MAE**	**R**^**2**^	**MAE**	**R**^**2**^	**MAE**	**R**^**2**^	**MAE**
LLR	ECFP4	0.513	0.495	0.623	0.439	0.657	0.415	0.867	0.222
	FCFP4	0.481	0.513	0.604	0.457	0.640	0.435	0.818	0.299
	MACCS	0.476	0.511	0.568	0.458	0.610	0.431	0.833	0.282
	DES	0.459	0.525	0.594	0.451	0.633	0.428	0.824	0.259
	LLR_consensus	0.608	0.420	0.711	0.371	0.743	0.354	0.908	0.186
SA	ECFP4	0.519	0.498	0.610	0.468	0.634	0.457	0.731	0.400
	FCFP4	0.492	0.513	0.593	0.481	0.619	0.470	0.716	0.438
	MACCS	0.484	0.517	0.573	0.485	0.608	0.470	0.745	0.397
	DES	0.472	0.522	0.576	0.477	0.610	0.462	0.745	0.361
	SA_consensus	0.577	0.452	0.664	0.424	0.690	0.416	0.779	0.365
SR	ECFP4	0.551	0.475	0.650	0.438	0.676	0.424	0.834	0.319
	FCFP4	0.520	0.495	0.627	0.458	0.656	0.444	0.772	0.391
	MACCS	0.494	0.510	0.585	0.476	0.621	0.460	0.765	0.381
	DES	0.473	0.521	0.576	0.477	0.610	0.461	0.747	0.360
	SR_consensus	0.593	0.442	0.683	0.411	0.710	0.402	0.825	0.330
GP	ECFP4	0.587	0.436	0.684	0.391	0.709	0.375	0.928	0.161
	FCFP4	0.547	0.462	0.659	0.413	0.694	0.394	0.844	0.278
	MACCS	0.489	0.493	0.585	0.451	0.630	0.428	0.845	0.292
	DES	0.501	0.494	0.610	0.445	0.647	0.426	0.817	0.297
	GP_consensus	0.623	0.413	0.717	0.374	0.744	0.362	0.916	0.213
Final_consensus		0.619	0.422	0.712	0.385	0.740	0.374	0.885	0.265

The improvement can be illustrated with the prediction of the following test compound. Flocoumafen (cas: 90035-08-8) is an anticoagulant rodenticides [13] with a LD_50_ of 6.336. As shown in Table 3, the neighbors selected from the reference set I have low similarities to the compound, and the resultant prediction errors are large. However, the similarities of the neighbors selected from the reference set II to flocoumafen are significantly increased, and the toxicity range of neighbors (the range is from 4.762 to 6.515) is also closer to the test compound. With these similar reference compounds, the results of Group II had significant improvement compared to those of Group I. Apparently, the reference set should be expanded not only in terms of chemical diversity but also their activity distribution to afford higher prediction accuracy of LLL models.

**Table 3:** The results of the test compound flocoumafen (cas: 90035-08-8) and its neighbors from the reference set I and II

	**Structure**	**Exp. LD**_**50**_^**a**^	**Dis.**^**b**^	**Pred. LD**_**50**_^**c**^	**Pred. LD**_**50**_^**d**^
Flocumafen		6.336	---	---	---
The neighbors from reference set I		4.068	0.543	3.435	3.085
		3.129	0.691		
		2.687	0.714		
		3.147	0.720		
		3.166	0.743		
The neighbors from reference set II		6.218	0.169	6.295	5.948
		5.807	0.177		
		5.743	0.194		
		5.686	0.338		
		5.873	0.343		

^a^Experimental –log(LD_50_).

^b^The Tanimoto distance of Flocumafen to its neighbor using ECFP4.

^c^The predicted –log(LD_50_) value by the best individual model.

^d^The predicted –log(LD_50_) value by the final consensus model.

### Effects of applicability domain

**Figure 3:**
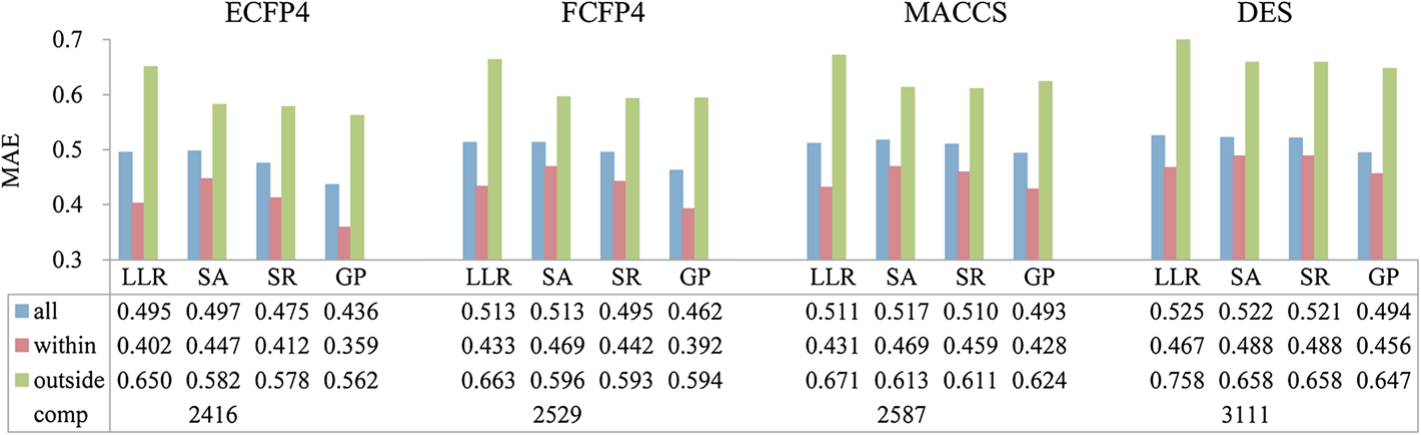
**The MAEs of all individual models for the test set in Group II.** all (blue): the MAEs of all compounds in the test set; within (red): the MAEs of compounds within AD; outside (green): the MAEs of compounds outside of the AD; comp: the number of compounds within the AD.

A large reference set with more similar compounds can improve the prediction performance of models. However, we still need to determine in which range and to what an extent the LLL models can be reliably applied. In this study, the AD is defined as a Tanimoto (or Euclidean) distance threshold between the test compound and its nearest neighbor. If the calculated distance is beyond the threshold, the prediction of the test compound is considered to be unreliable. Through the Equation (7), we obtained the AD threshold of every individual model. In Group I, the distance thresholds are 0.595 for ECFP4, 0.477 for FCFP4, 0.257 for MACCS, and 0.035 for DES. In Group II, the distance thresholds are 0.498 for ECFP4, 0.378 for FCFP4, 0.195 for MACCS, and 0.025 for DES. Obviously, the expansion of the reference dataset made it possible to find more similar compounds for the test compound, and hence enlarged the model AD. In order to investigate the influence of AD on prediction error, we further applied the models of Group II to the test compounds within and outside AD, respectively. From the distribution of MAEs (see Figure 3), we can find that the MAEs follow the trend within < all < outside in all individual models, which is in line with our initial hypothesis that AD delimitation is required to assess the reliability of a prediction. Specifically, the models using ECFP4 have the best performance for compounds both within and outside AD, but have the smallest AD. In contrast, the models using “DES” have the worst performance but the largest AD. When using the same similarity metrics, GP in most cases showed the best performance among the four LLL model types, while the other three exhibited some differences for compounds within and outside the AD. For example, the MAEs of LLR are lower than SA and SR for compounds within AD, but are higher for those outside AD. All these observations suggested that the individual LLL models explain complementary portions of the variance in chemicals’ LD_50_ data, which also account for the improvement in consensus modeling in this study.

As the final consensus model is constructed by taking the arithmetic average of all LLL models, its reliability to predict a test compound highly depends on its constituent models. Therefore, the “consensus prediction fraction” (i.e., the ratio of individual models being reliable to predict a new compound), is used to define the AD of the final consensus model [7]. In the current study, the final model comprises four kinds of individual LLL models, and if one of them can reliably predict a new compound, the “consensus prediction fraction” is 25% for this compound. Only if the “consensus prediction fraction” is larger than or equals to a predefined threshold, the final consensus model is considered as reliable. When the threshold is set to be 75%, there are totally 2,378 test compounds within the AD, on which the final consensus model has significantly improved performance. For example, for the final consensus model of Group II, the MAEs of all, within, and outside are 0.422, 0.358, and 0.523, respectively. Obviously, the application of AD can tell us when the final consensus model provides a reliable and better estimation of acute oral toxicity in rat.

## Experimental

### Datasets

The rat LD_50_ data by oral exposure were collected from Zhu *et al.*[7], United States Environmental Protection Agency dataset [11], and Accelrys Toxicity database 2011.4 [12]. The final dataset included 9,617 compounds after removing the duplicated and wrong structures. Among them, there are 3,472 and 3,874 compounds identical to Zhu’s training set and test set, which will be used as the reference Set I and the test set, respectively. For comparison, we prepared three subsets representing different prediction coverage, in which the test compounds were ranked ascendingly according to their distance to their nearest neighbors, and then the first 2,896, 2,583 and 743 compounds were selected, respectively, to comprise multiunit test sets (hereafter called “Set_2896”, “Set_2583”, “Set_743”). In addition we also constructed an expanded reference set named the reference set II, which contains 5,743 compounds including the whole reference Set I, the compounds from EPA dataset and Accerlys Toxicity dataset. The original unit of LD_50_ was firstly converted to -log(mol/kg) to conform to the standard QSAR practice.

### Feature sets and similarity measurement

The initial structures of all compounds were optimized by Sybyl 6.8 [14] which used the Powell method with Tripos Force fields and Gasteiger–Hückel charges. Further structural optimization was performed using the AM1 semi-empirical method implemented in AMPAC 8.16 [15]. To measure the similarity between compounds, we tried both physicochemical descriptors and molecular fingerprints. For the former, totally 490 descriptors were calculated with Codessa 2.7.2 [16] and Discovery Studio 2.5 [17]. After removing those descriptors with zero variance or that cannot be calculated for some compounds, altogether 286 descriptors were remained (hereafter called “DES”). For any two compounds, their normalized Euclidean distance (*Dis*) [18] was defined below:

(1) $$\mathrm{Dis}=\frac{\sqrt{\sum_{i=1}^{286}{\left(X_{\mathrm{iA}}-X_{\mathrm{iB}}\right)}^{2}}}{286}$$

where *X*_*iA*_ and *X*_*iB*_ are the normalized values of the *i*-th descriptor of *A* and *B*, respectively. For molecular fingerprints, the widely used ECFP4 [19], FCFP4 [19], and MACCS [20] were generated with RDKit [21]. Given the bit vectors of those fingerprints, the Tanimoto similarity (*Sim*) [18] between any two compounds were computed, and their Tanimoto distance (Dis’) was given by the following transformation:

(2) $$\mathrm{Di}s^{'}=1-\mathrm{Sim}$$

### Prediction models

For each given query compound, four sets of *k* nearest neighbors were retrieved from the reference set using different feature sets. Then local lazy learning strategies were applied to construct local models, from which consensus model was built. All the computation was done using in-house C/Python programs.

## Conclusion

In this study, four kinds of local lazy learning schemes were combined with four similarity metrics to predict the acute toxicity in rat. Different from the conventional global QSAR models built upon the entire diverse data set, these LLL models were constructed “on-the-fly” by only utilizing the analogical compounds of a query. Accordingly, the detailed and subtle local structure-toxicity relationships around the query compound can be captured, which might be otherwise overshadowed by the large amount of employed training compounds in global models. As the approach relies on a priori knowledge about the toxicity profile of a query’s neighbors, its prediction accuracy can be improved by enriching the size and the structural diversity of the reference set, and the “on-the-fly” feature of LLL models also allows for a timely update and expansion. To reduce the high variance of individual LLL models, a consensus modeling scheme was employed, which further improved the accuracy of LD_50_ prediction. For the “Set_2896”, the R^2^ of the final consensus model using reference set II was enhanced from 0.545 to 0,712, and the MAE of prediction was reduced to 0.385. Moreover, by introducing the concept of AD, the reliability of a predication can be evaluated. For the compounds within AD, their toxicity can be more accurately predicted. The outstanding performance of our approach suggests that LLL models are feasible and effective for in silico prediction of acute oral toxicity in rat. We expect this method would also be a useful tool to provide inspiration for discovering novel drug candidates with favorable safety profile.

## Methods

### kNN-based LLL Models

Four sets of *k* nearest neighbors were provided for a test compound by ECFP4, FCFP4, MACCS, and DES. For each set, four types of LLL models were built, with details described as follows:

**Figure 4:**
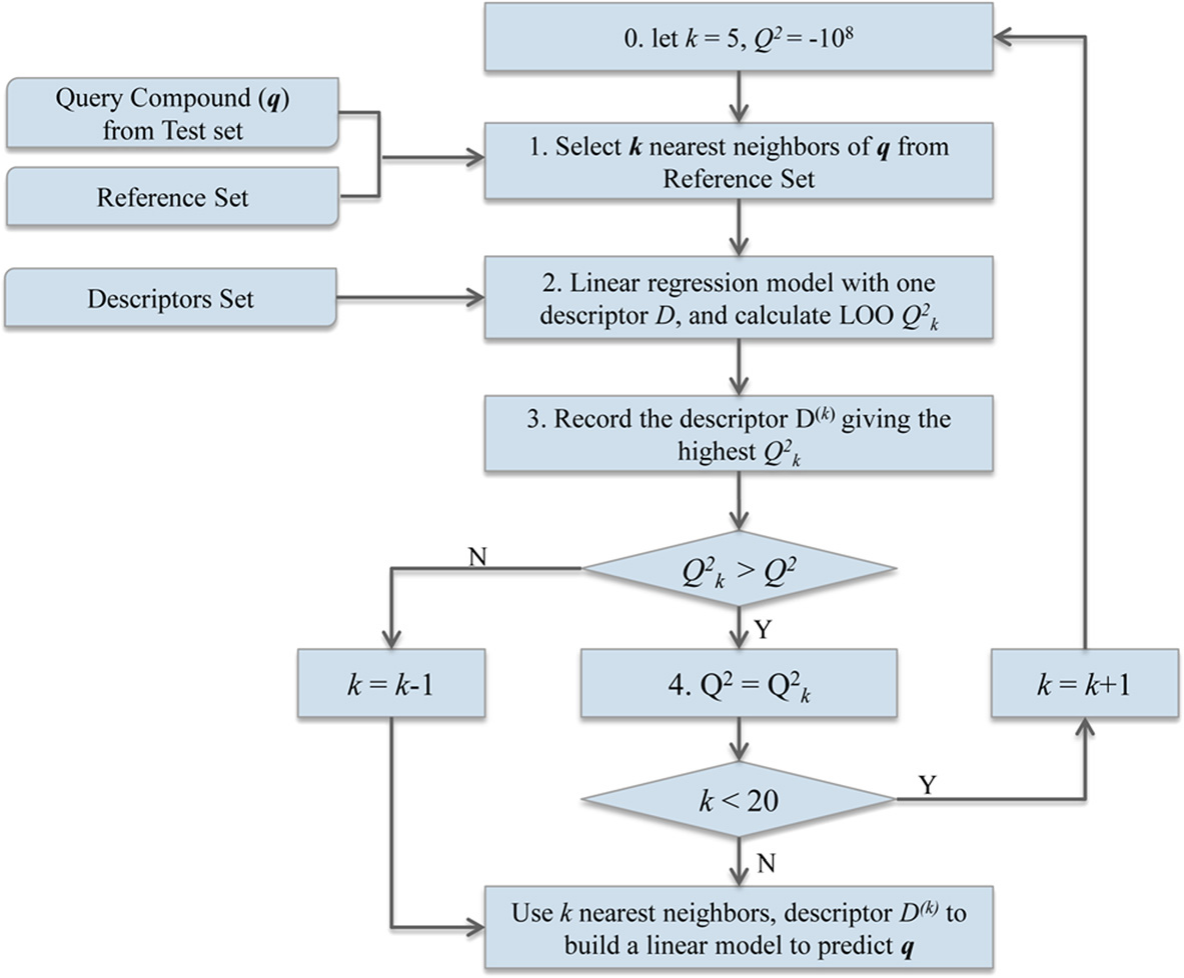
The flowchart of the LLR modeling.

**Figure 5:**
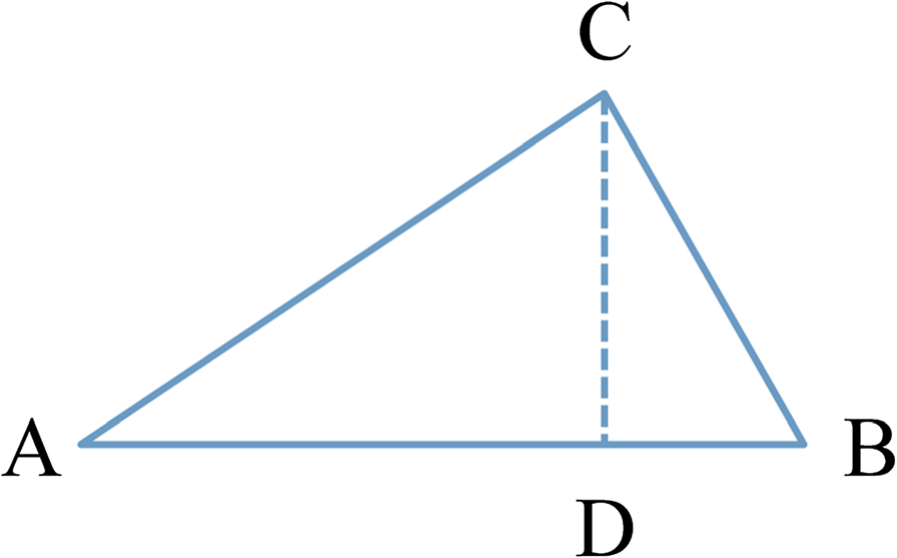
**Triangle inequality.** An triangle can be constructed for any 3 compounds by using Euclidean or Tanimoto distance as the length of edge. *D* is the projection of vertex *C* on line *AB*.

a. **Local lazy regression (LLR)**: For each test compound, only one most relevant descriptor was selected to build a linear equation based on *k* nearest neighbors. During the procedure, both the descriptor and the number of nearest neighbors were optimized. Initially let *k* = 5 and *Q*^*2*^ = −10^8^ (an arbitrary negative value that will be updated during the iteration), the algorithm was described as follows: (1). Select *k* nearest neighbors of the query compound (*q*) from the reference set. (2). Use one single descriptor to build a linear regression model, and perform a leave-one-out (LOO) cross-validation for the model. Note here the descriptor value calculated for the query was compared with those calculated for its neighbors. If the query’s value falls outside the range of its neighbors, the descriptor was disregarded to avoid yielding an extrapolated value. (3). After traversing all descriptors, record the descriptor *D*^(*k*)^ that leads to the highest LOO *Q*^*2*^_*k*_. (4). If *Q*^2^_*k*_ > *Q*^2^, update *Q*^2^ and *D*^*(k*)^, then add one more neighbor, and repeat steps 1–3 until *k* > 20; otherwise, the iteration is terminated, and let *k* = *k*-1. Finally, the query compound was predicted by a linear model using *k* nearest neighbors and the descriptor *D*^*(k)*^. Figure 4 shows the flowchart of the LLR modeling.

b. **SA model**: The predicted toxicity of the test compound is calculated as [22,23]:

(3) $$y_{\mathrm{pre}}=\frac{\sum_{i=1}^{n}y_{i,\mathrm{obs}}}{n}\;$$

where *y*_*i,obs*_ is the experimental value of the *i*-th neighbor, and *y*_*pre*_ is the predicted value of the test compound. The value of *n* was determined by using 10-fold cross validation of the reference set.

c. **SR model**: The predicted toxicity of the test compound is calculated by [22,23]:

(4) $$y_{\mathrm{pre}}=\sum_{i=1}^{n}\left\{\frac{S_{i}}{\sum_{j=1}^{n}S_{j}}*y_{i,\mathrm{obs}}\right\}$$

where *s*_*i*_ is the similarity value between the test compound and the *i*-th neighbor. The value of *n* was determined in the same way as being used to build the SA model.

d. **GP model:** For a query compound *C* and any two neighbors *A* and *B*, a triangle can be constructed in a multidimensional descriptor space (Euclidean space). As shown in Figure 5, the value of projection point *D* is used as an estimate of *C*, and under the assumption that the y-values is linearly changed along the line *AB*, the value of *D* can be calculated as follows:

(5) $$\left\{\begin{array}{l} y_{D}=y_{A}-\frac{d_{\mathrm{AD}}}{d_{\mathrm{AB}}}*\left(y_{A}-y_{B}\right)\;\mathrm{if}\;d_{\mathrm{AB}}\neq 0\\ y_{D}=\frac{y_{A}+y_{B}}{2}\;\mathrm{otherwise} \end{array}\right)\;$$

where *d*_*AB*_ is the distance between neighbor *A* and *B*, *d*_*AD*_ *= d*_AC_* cos∠CAB, and *y* is –log(LD_50_) value of compound. Besides, as being proved by Lipkus [24] that Tanimoto distance from bit vectors also satisfies the triangle inequality, the GP model can also be applied to those using Tanimoto distance as a similarity measurement. Given *k* nearest neighbors of the query, any pair of the neighbors can build a GP model to yield a projection point *D*. We take the weighted average of all $C_{2}^{k}$ individual estimates of the projection points as the final prediction:

(6) $$y_{\mathrm{pre}}=\frac{\sum S_{\mathrm{AC}}\cdot S_{\mathrm{BC}}\cdot y_{D}}{\sum S_{\mathrm{AC}}\cdot S_{\mathrm{BC}}}$$

where *S* is the similarity between a pair of neighbors, *y*_*D*_ is obtained by Equation (5). Of note the above definitions assume that none of the edges of a triangle is degenerated. If a query compound has a neighbor with a zero distance, its LD_50_ is directly estimated by that neighbor. Moreover, a proper *k* was automatically optimized for each test compound by applying the same strategy showing in Figure 4. This procedure is similar to that of LLR except that the initial *k* value was set to 3.

### Consensus model

The LLL methods in this study are all similarity based, of which the decision boundary or value largely depends on the input points and their particular positions. However, individual models using different modeling methods or similarity measurements could vary significantly and capture different part of the relationship. To reduce the high variance, consensus model can be established by combining each individual model, which has been demonstrated to be an effective means to improve the performance of similarity based methods [7,25-29]. In this study, the strategy as used in Zhu *et al.*[7] was applied to build consensus model, in which the predicted toxicity for each compound equals to the arithmetical mean of all predicted values of individual models. For each type of LLL method, a consensus model was constructed by averaging the results using different similarity metrics, named as “LLR_consensus”, “SA_consensus”, “SR_consensus”, and “GP_consensus”, respectively. Besides, a consensus model named “Final_consensus” was built by averaging all the 16 individual models.

### Applicability domain (AD)

In the QSAR models, the AD defines the chemical space for which the model is considered to be applicable [30]. Here we use the distance-based AD definition [30,31], which assumes a prediction reliable if the concerned molecule is located in the neighborhood of the reference set compounds. The threshold of distance *D*_*T*_ is defined as:

(7) $$D_{T}=\bar{d}+Z*\sigma$$

Where $\bar{d}$ is the average Tanimoto or Euclidean distance between all compound and their nearest neighbor in the reference set, *σ* is the standard deviation of these distances, and *Z* is an arbitrary parameter to control the threshold level (here set to 0.5). If the distance of the compound to its nearest neighbor exceeds this threshold, this test compound is treated as an “outlier”, and the prediction result is considered to be unreliable.

## Abbreviations

LD_50_: Median lethal dose; LLL: Local lazy learning; LLR: Local lazy regression; QSTRs: Quantitative structure-toxicity relationships; LOO: Leave-one-out; AD: Applicability domain; *k*NN: *k* nearest neighbors; MAE: Mean absolute error.

## Competing interests

The authors declare that they have no competing interests.

## Authors’ contributions

Conceived and designed the experiments: MYZ and XML. Performed the experiments: JL, JLP, JAW, and QCS. Analyzed the data: JL, JLP, YB, MYZ, and XML. Wrote the paper: JL, JLP, MYZ, and XML. All authors discussed the results and commented on the manuscript. All authors have given approval to the final version of the manuscript.

## Supplementary Material

Additional file 1 Frequency analysis of fingerprints to find out potential substructures causing acute toxicity. Click here for file
