# Supplementary material for: Estimation of acute oral toxicity in rat using local lazy learning
Source: J Cheminform. 2014 May 16;6:26. doi: 10.1186/1758-2946-6-26 (PMC4047767; doi:10.1186/1758-2946-6-26)
Supplement: Additional file 1 — Frequency analysis of fingerprints to find out potential substructures causing acute toxicity. [file 1758-2946-6-26-S1.docx]

Estimation of acute oral toxicity in rat using local lazy learning

Jing Lu, Jianlong Peng, Jinan Wang, Qiancheng Shen, Yi Bi, Likun Gong, Mingyue Zheng^*^, Xiaomin Luo^*^, Weiliang Zhu, Hualiang Jiang, Kaixian Chen

Analysis of privileged substructures from ECFP4 fingerprints

In this study, different LLL strategies were designed to give a quantitative estimation of acute oral toxicity in rat, among which LLL models combined with ECFP4 showed the best predictability. It is therefore of interest to investigate which ECFP4 fingerprints show positive contribution and negative contribution to the toxicity. Recently, Li et al. [1] published a related work, in which they constructed multi-classification models using two types of fingerprints, MACCS keys and FP4 fingerprint, as features. Frequency analysis was applied to find out privileged substructures that more commonly present in chemical classes showing toxicity.

Here, the strategy proposed by Li et al. [1] was applied to our reference set II to give a simple frequency analysis of each ECFP4 fingerprint, which can be briefly described as follows. Firstly, according to the data separation rule used in the reference [1], our reference set II was divided into four categories, each of which contains 717, 1461, 2787, and 778 compounds, respectively. Then, a frequency value as defined below was calculated for each fragment:

$F=\frac{N_{fragment\_class}/N_{class}}{N_{fragment\_total}/N_{total}}$ (S1)

where *N_fragment_class_* is the number of compounds containing the fragment in category I and II chemicals, *N_class_* if the number of category I and II chemicals, *N_fragment_total_* is the total number of compounds containing the fragment, *N_total_* is the total number of compounds in our reference set. Some of fragments being enriched in category I or II were listed in Table S1. When compared to the results reported by Li et al., some of privileged substructures could also be found in our reference set, e.g. the phosphonic acid, phosphonic acid derivatives, alkyfluoride, nitrile, chloroalkene, and carbamate etc. Compared with MACCS and FP4 fingerprint used by Li et al., ECFP4 is a type of circular topological fingerprints that are not predefined and can represent a huge number of different molecular features. Accordingly, we are able to analyze more fragments, and some of them are not found in the work of Li et al. For example, 2-(trifluoromethyl)-benzimidazole (No. 6 in Table S1) could be directly obtained from ECFP4 fingerprints, while Li et al. only found a simple alkylfluoride from the predefined fingerprint dictionary, which needs further manual checking to accurately define the fragment. In addition, Aziridines (No. 8 in Table S1) was found to be highly enriched in category I compounds, which is electrophiles and able to form adducts with DNA. Previous study has been reported that ethyleneimine (CAS No. 151-56-4) could induced renal papillary necrosis in rats [2].

Table S1. Some examples of privileged fragments

| No. | Fragment ^a^ | Frequency in each category | | | |
| --- | --- | --- | --- | --- | --- |
|  |  | I | II | III | IV |
| 1 | 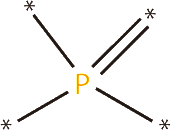 | 3.398 | 1.357 | 0.401 | 0.247 |
| 2 | 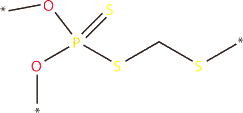 | 5.780 | 1.081 | 0.000 | 0.000 |
| 3 | 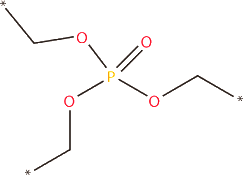 | 3.559 | 1.112 | 0.499 | 0.204 |
| 4 | 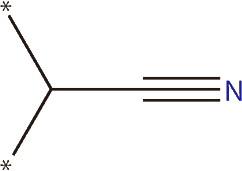 | 1.101 | 2.364 | 0.389 | 0.521 |
| 5 | 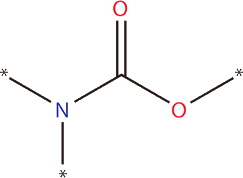 | 2.960 | 1.393 | 0.468 | 0.344 |
| 6 | 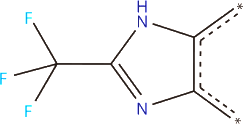 | 6.652 | 0.653 | 0.000 | 0.000 |
| 7 | 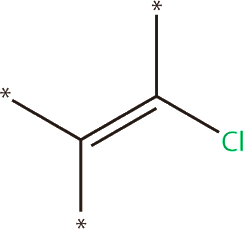 | 2.240 | 1.663 | 0.532 | 0.272 |
| 8 | 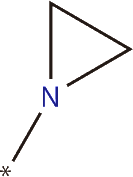 | 4.298 | 1.808 | 0.000 | 0.000 |

^a^ * indicates matching any atom.

**Reference**

1. Li, X, Chem, L, Chem, FX, Wu ZR, Bian HP, Xu CY, Li WH, Liu GX, Shen X, Tang Y: **In silico prediction of chemical acute oral toxicity using multi-classification methods**. *J Chem Inf Model* 2014. DOI: 10.1021/ci5000467
2. Ellis BG, Price RG: **Urinary enzyme excretion during renal papillary necrosis induced in rats with ethyleneimine.** *Chem Biol Interact* 1975, **11**:473-482.
